# The complete chloroplast genome of *Phlegmariurus phlegmaria*, one representative species of genus *Phlegmariurus*

**DOI:** 10.1080/23802359.2020.1820392

**Published:** 2020-09-29

**Authors:** Li-Ming Tang, Ri-Hong Jiang, Jia-Cheng An

**Affiliations:** a Forestry Department of Guangxi, Nanning, PR China; b Key Laboratory of Central South Fast-growing Timber Cultivation of Forestry Ministry of China, Guangxi Key Laboratory of Superior Timber Trees Resource Cultivation, Guangxi Forestry Research Institute, Nanning, PR China

**Keywords:** *Phlegmariurus phlegmaria*, chloroplast genome, Lycopodiaceae, lycophytes phylogeny

## Abstract

*Phlegmariurus* (Herter) Holub is the largest genus of Lycopodiaceae, with about 250 species distributed in the tropics and subtropics of the world. *Phlegmariurus phlegmaria* is the representative species of *Phlegmariurus*. In this study, we reported the complete chloroplast genome of *P. phlegmaria*. This complete chloroplast genome is 1,49,711 bp in size. In total, 134 genes were identified, including 84 protein-coding genes, 42 tRNA genes, and eight rRNA genes. In phylogenetic analysis, a close relationship with genus *Huperzia* was supported by maximum-likelihood (ML) tree. The complete plastome of *P. phlegmaria* will provide potential genetic resources to understand the evolution of lycophytes.

*Phlegmariurus* (Herter) Holub is the largest genus of Lycopodiaceae, with about 250 species distributed in the tropics and subtropics of the world (PPGI 2016). *Phlegmariurus* is mostly closely related to the temperate genus *Huperzia* (Wikström and Kenrick 2000; Field et al. 2016), and these two genera are belonging to Lycopodiaceae subfamily Huperzioideae (Wagner and Beitel 1992; Øllgaard 2015; PPGI 2016). *Phlegmariurus phlegmaria* is the representative species of genus *Phlegmariurus*. Here, we assembled and characterized the complete plastome of *P. phlegmaria*. It will provide great genetic resources to amplify the relationship between *Phlegmariurus* and *Huperzia*, and understand the phylogeny relationship of lycophytes.

The plant material of *P. phlegmaria* was collected from city Pingxiang (Guangxi, China; 22.09°N, 106.76°E). Voucher specimen (9461) and DNA sample were deposited in the herbarium of Institute of Botany, CAS (PE). In total, 6G high-quality clean reads (150 bp PE read length) were generated with adaptors trimmed. The Get Organelle (Jin et al. 2018), Bandage (Wick et al. 2015), GeSeq (Tillich et al. 2017), were used to align, assemble, and annotate the chloroplast genome.

The full length of *P. phlegmaria* chloroplast genome (GenBank Accession No. MT786212) was 1,49,711 bp and comprised a large single-copy region (LSC with 99,862 bp), a small single-copy region (SSC with 19,465 bp), and two inverted repeat regions (IR with 15,192 bp). The plastid genome length of *P. phlegmaria*is similar to *P. carinatus* and *Huperzia javanica* (Zhang et al. 2017; Luo et al. 2019). The overall GC content of *P. phlegmaria* cp genome was 33.8%, in particular 31.4% in the LSC, 44.0% in the IR, 30.1% in the SSC region. A total of 134 genes were contained in the chloroplast genome, including 84 protein-coding genes (*nad5* and *ndhF* genes were duplicated in the repeat region), 42 tRNA genes (*trnA-UGC*, *trnE-UUC*, *trnI-GAU*, *trnN-GUU*, *trnR-ACG*, and *trnV-GAC* tRNA genes were duplicated in the repeat region), and eight rRNA genes (*rrn16*, *rrn23*, *rrn4.5*, and *rrn5* genes were duplicated in the repeat region).

Ten complete chloroplast genomes were selected to infer the phylogenetic relationships among the main representative species of lycophytes with *Marchantia paleacea* (liverwort) as the out group. All of these ten complete chloroplast sequences were aligned by the MAFFT version 7 software (Katoh and Standley 2013) and trimmed by TrimAl (Capella-Gutierrez et al. 2009). A maximum-likelihood (ML) tree was inferred by Ultrafast bootstrapping with 1000 replicates through IQ-TREE version 1.5.5 (Nguyen et al. 2015) based on the TVM + F + R3 nucleotide substitution model, which was selected by ModelFinder (Kalyaanamoorthy et al. 2017). The result revealed that the genus *Phlegmariurus* is strongly supported as monophyletic and sister to the genus *Huperzia*. (Figure 1).

**Figure 1.**
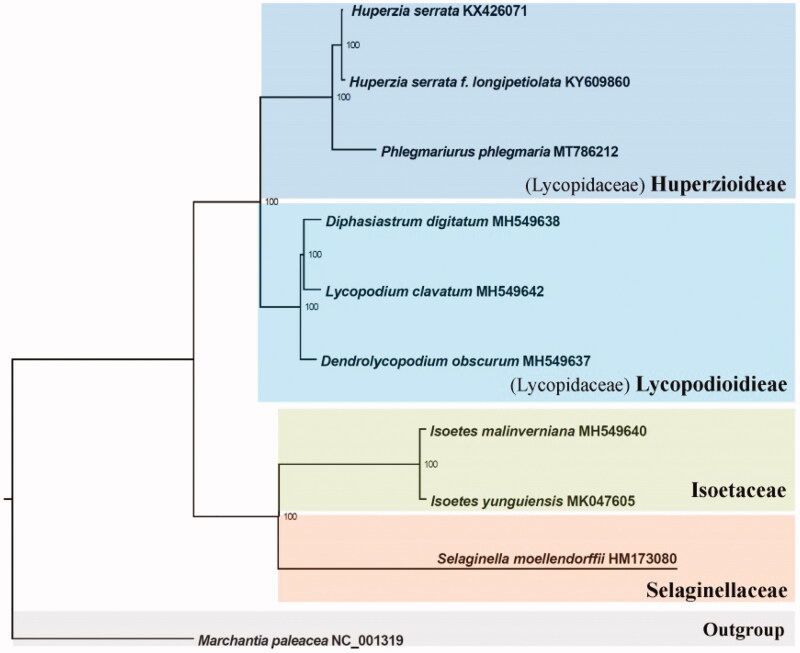
The ML phylogeny results from ten complete plastome sequences by IQ-TREE. Accession numbers: *Huperzia serrata* KX426071, *H. serrata f. longipetiolata*, KY609860, *Phlegmariurus phlegmaria* MT786212, *Diphasiastrum digitatum* MH549638, *Lycopodium clavatum* MH549642, *Dendrolycopodium obscurum* MH549637, *Isoetes malinverniana* MH549640, *Isoetes yunguiensis* MK047605, *Selaginella moellendorffii* HM173080, and *Marchantia paleacea* NC_001319.

## Data Availability

The complete chloroplast genome sequence of *Phlegmariurus phlegmaria* in this study was submitted to the NCBI database under the accession number MT786212. https://www.ncbi.nlm.nih.gov/nuccore/?term=MT786212
